# LIN7A Depletion Disrupts Cerebral Cortex Development, Contributing to Intellectual Disability in 12q21-Deletion Syndrome

**DOI:** 10.1371/journal.pone.0092695

**Published:** 2014-03-21

**Authors:** Ayumi Matsumoto, Makoto Mizuno, Nanako Hamada, Yasuyuki Nozaki, Eriko F. Jimbo, Mariko Y. Momoi, Koh-ichi Nagata, Takanori Yamagata

**Affiliations:** 1 Department of Pediatrics, Jichi Medical University, Tochigi, Japan; 2 Department of Molecular Neurobiology, Institute for Developmental Research, Aichi Human Service Center, Kasugai, Japan; CNRS UMR7275, France

## Abstract

Interstitial deletion of 12q21 has been reported in four cases, which share several common clinical features, including intellectual disability (ID), low-set ears, and minor cardiac abnormalities. Comparative genomic hybridization (CGH) analysis using the Agilent Human Genome CGH 180K array was performed with the genomic DNA from a two-year-old Japanese boy with these symptoms, as well as hypoplasia of the corpus callosum. Consequently, a 14 Mb deletion at 12q21.2-q21.33 (nt. 77 203 574–91 264 613 bp), which includes 72 genes, was detected. Of these, we focused on *LIN7A*, which encodes a scaffold protein that is important for synaptic function, as a possible responsible gene for ID, and we analyzed its role in cerebral cortex development. Western blotting analyses revealed that Lin-7A is expressed on embryonic day (E) 13.5, and gradually increases in the mouse brain during the embryonic stage. Biochemical fractionation resulted in the enrichment of Lin-7A in the presynaptic fraction. Suppression of Lin-7A expression by RNAi, using *in utero* electroporation on E14.5, delayed neuronal migration on postnatal day (P) 2, and Lin-7A-deficient neurons remained in the lower zone of the cortical plate and the intermediate zone. In addition, when Lin-7A was silenced in cortical neurons in one hemisphere, axonal growth in the contralateral hemisphere was delayed; development of these neurons was disrupted such that one half did not extend into the contralateral hemisphere after leaving the corpus callosum. Taken together, *LIN7A* is a candidate gene responsible for 12q21-deletion syndrome, and abnormal neuronal migration and interhemispheric axon development may contribute to ID and corpus callosum hypoplasia, respectively.

## Introduction

Interstitial deletion of 12q21 has been reported in four cases since 1999 [1]–[4]. Common clinical features of this deletion include: intellectual disability (ID), low-set ears, sparse hair, prominent forehead, hyper- or hypo-telorism, minor cardiac abnormalities, and cutaneous findings, such as hyperkeratotic papular eruption and atopic dermatitis. These phenotypes resemble those in cardiofaciocutaneous (CFC) syndrome, which is characterized by a distinctive facial appearance, heart defects, and ID [5]. However, while genes on the RAS-MAPK pathway have been identified as the causative genes for CFC syndrome, no genes related to this signaling pathway are located within the reported deleted regions of 12q21.

Outside of the RAS-MAPK pathway, many genes that are related to synaptic function and plasticity have been reported as responsible for ID [6], [7]. Lin7 – the product of the *LIN7* gene – is known to play a crucial role in synapse functions. Lin7 is a small scaffold protein containing a L27 and a PDZ domain at the N- and C-terminus, respectively. The L27 domain mediates heterodimerization with several membrane-associated guanylate kinase (MAGUK) proteins – including calcium/calmodulin-dependent serine protein kinase (CASK), protein associated with LIN-7 (Pals), synapse-associated protein 97 (SAP97), and PSD95/93 – which form the core of the protein complexes that mediate synaptic development, plasticity, and functionality [8]. On the other hand, the PDZ domain (corresponding to the first letters of PSD-95, Discs-large, and ZO-1) binds with many proteins, which are essential for neuronal cell polarity, cell adhesion, and cell signaling [9].

Accumulating evidence has uncovered a possible relationship of *LIN7* with neuronal disorders. Vertebrates have three *LIN7* isoforms, *LIN7A–C*, also known as *MALS/Veli1–3* [10], [11]. Recently, polymorphisms of *LIN7* have also been shown to be associated with attention-deficit/hyperactivity disorder (ADHD) [12], and microdeletions at 11p14.1 (where *LIN7C* is localized) are reportedly associated with ADHD, autism, and developmental delay [13]. Furthermore, decreased Lin-7B expression in pyramidal neurons in cerebral cortex layer V may contribute to impaired neuronal connectivity in Huntington's disease [14].

The present study describes a Japanese boy who presented facial dysmorphism and ID, similar to previously reported patients with 12q21 deletion. Using array comparative genomic hybridization (CGH) analysis, we identified a deletion at 12q21.2–q21.33, which is the narrowest deletion found in a patient with 12q21-deletion syndrome to date. Among the genes located in the deleted region, we focused on *LIN7A*, performing biochemical and RNAi analyses to clarify the pathophysiological significance of Lin-7A in 12q21-deletion syndrome. While Lin-7A did not appear to be involved in neuronal cell proliferation, we determined that loss of Lin-7A function induced defective migration and axonal growth of excitatory pyramidal neurons during corticogenesis. Thus, functional defects of Lin-7A are likely to cause defective cortical development, leading to ID in 12q21-deletion syndrome.

## Materials and Methods

### Ethics statement

This study was approved by the bioethics committee for human gene analysis at Jichi Medical University (approval number: 11–14). Written informed consent was obtained from the mother for the child and herself, and from the father for himself. We followed the Fundamental Guidelines for Proper Conduct of Animal Experiment and Related Activities in Academic Research Institutions under the jurisdiction of the Ministry of Education, Culture, Sports, Science and Technology, and all of the protocols for animal handling and treatment were reviewed and approved by the Animal Care and Use Committee of Institute for Developmental Research, Aichi Human Service Center (approval number, M10).

### Patient report

The patient was a boy of two years and 11 months of age. He was the first child born to a non-consanguineous and healthy 24-year-old mother and 26-year-old father. The patient was born by cesarean section at 38 weeks and three days. At birth, his height, weight, and occipito-frontal circumference were 45.6 cm (1.64 SD), 2330 g (1.40 SD), and 32.3 cm (0.60 SD), respectively. His growth parameters were normal.

**Figure 1:**
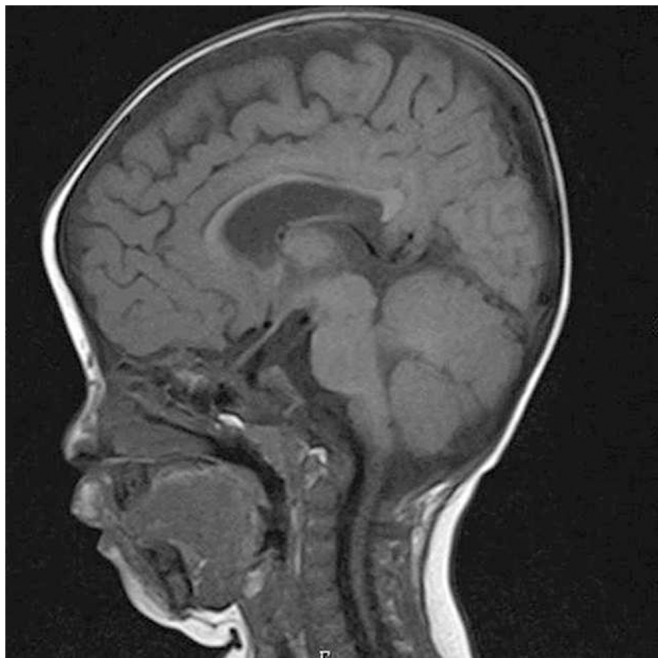
Cranial MRI findings at 10 months of age. Sagittal and T1-weighted image, showing mild hypoplasia in the corpus callosum.

At two years and 11 months of age, the boy's developmental quotient (DQ) was 20, according to the kinder infant development scale (KIDS). He could sit without support but could not walk or speak any meaningful words. He showed prominent forehead, hypotelorism, short upturned nose, long philtrum, small mandible, high-arched palate, low-set ears, sparse hair, and internal strabismus. He also had mild spastic diplegia, and his neurological evaluation revealed hyperreflexia of the patellar and Achilles tendon reflexes. Cranial magnetic resonance imaging (MRI) showed ventriculomegaly and mild hypoplasia of the corpus callosum (Figure 1).

### Array CGH analysis

After obtaining blood from the patient and his parents, lymphocytes were extracted, and lymphoblasts were established by Epstein–Barr virus. Array CGH analysis was performed using the Agilent Human genome CGH 180K (Agilent Technologies, Santa Clara, CA, USA). In brief, DNA was isolated from the lymphocytes, using the DNeasy Blood and Tissue kit (Qiagen, Hilden, Germany), according to the manufacturer's instructions. Genomic DNA (1 μg) from the patient and reference DNA samples from unaffected volunteers (Coriell Institute for Medical Research, Camden, New Jersey, USA) were labeled with fluorescent dyes (Cy5-dUTP and Cy3), hybridized at 65 °C for 24 hours, and then subjected to the array. The hybridized array was scanned with an Agilent DNA Microarray Scanner (Agilent Technologies). The resulting images were analyzed by quantifying the Cy3 and Cy5 fluorescence intensity at each feature on the array, using the Agilent Feature Extraction Software (Agilent Technologies). Finally, the data were calculated using the Agilent Workbench Software (Agilent Technologies).

### Plasmids

Human LIN7B-encoding cDNA was obtained as previously described [15]. Mouse Lin7A, Lin7B, and human Lin7A were obtained by PCR from brain cDNA pools, and ligated into pCAG-Flag, pCAG-Myc, or pCAG-GFP vectors. All constructs were verified by DNA sequencing.

### RNA interference

Modifications of the pSUPER-puro vector (OligoEngine, Seattle, WA, USA) were designed to target two distinct coding sequences in m*Lin7A*: pSUPER-mLin7A#1 targets 5′-GTGTATCAATACATGCATG-3′, 202–220; and pSUPER-mLin7A#2 targets 5′-GTTGAACTGCCAAAGACTG-3′, 325–343 (numbers indicate the position from the transcription start site). As an RNAi-resistant version of mLin7A, we used the human ortholog hLin7, in which the target sequence against pSUPER-mLin7A#1 contains a mismatched nucleotide (5′-GTGTATCAATATATGCATG-3′) as marked with an underline. The target sequences for pSuper-mLin7A#1 and #2 contain mismatched nucleotides when compared to the corresponding sequences in mLin7B (targets 5′-GTGTATGAACAGCTCTATG-3′, 5′-GTGGAACTACCGAAGACTG-3′), respectively, as marked with underlines.

### Cell culture and transfection

COS cells were cultured essentially as previously described [16]. Transient transfection was carried out using the Lipofectamine method (Invitrogen, Carlsbad, CA, USA).

### Primary antibodies

We generated polyclonal rabbit anti-Lin7 antibodies against affinity-purified bacterially synthesized human Lin-7B [15]. Characterization of anti-Lin7 was performed previously [15]. Polyclonal rabbit anti-GFP was prepared as described [17]. The mouse monoclonal antibody against glial fibrillary acidic protein (GFAP) was purchased from Chemicon (Temecula, CA, USA). Mouse monoclonal anti-β-tubulin, anti-Flag M2, and rabbit polyclonal anti-Flag were purchased from Sigma (Tokyo, Japan).

### Subcellular fractionation of rat brain

Subcellular fractionation of fresh adult rat brains was performed essentially as described previously [18].

### *In utero* electroporation

Pregnant ICR mice were purchased from SLC Japan (Shizuoka, Japan). *In utero* electroporation was performed essentially as described previously [19], [20]. Briefly, 2 μL of nucleotide solution containing expression plasmids and pSUPER-RNAi plasmid (2 μg each) was introduced into the lateral ventricles of embryos, followed by electroporation using a CUY21 electroporator (NEPA Gene, Chiba, Japan) with 50 ms of 30 V electronic pulses, six times with 950 ms intervals. It is most likely that the expression and RNAi plasmids were efficiently co-transfected into the majority of the neuronal progenitor/stem cells at the transfected region [19]. All electroporations were performed on embryonic day 14.5 (E14.5), and at least five brains were used for each experiment.

### Quantitative estimation of neuronal migration

The distribution of GFP-positive cells in brain slices was quantified, as follows: Coronal sections of cerebral cortices containing the labeled cells were classified into four regions: layers II–IV and V–VI, and the IZ and SVZ/VZ, as described previously [21]. The number of labeled cells (>100) in each region was calculated for least three slices per brain.

### EdU incorporation experiment

Embryos were electroporated *in utero* at E14 with the pCAG-EGFP vector and the pSUPER vector (control) or pSUPER-mLin7A#1. Thirty hours after electroporation, pregnant mice were given an intraperitoneal injection of EdU (25 mg/kg body weight). One hour after the injection, embryonic brains were fixed with 4% paraformaldehyde, and vibratome sections were made. Finally, GFP and EdU were detected with anti-GFP and Alexa Fluor555 azide (Life Technologies, Palo Alto, CA, USA), following the manufacturer's protocol.

### Statistical analysis

Results are expressed as mean ± s.e.m. When data were obtained from only two groups, Student's *t*-test was used for comparison. The rate of cell scores were initially analyzed using the one-way analysis of variance (ANOVA). Subsequently, a Fisher's least-significant difference (LSD) test was applied to absolute values as a *post-hoc* test of multiple comparisons. A *P* value of <0.05 was considered to indicate statistical significance. Statistical analysis was performed using Statview software (SAS Institute, Cary, NC, USA).

## Results

### Chromosomal analysis

The karyotype of the proband's amniotic cells was 46, XY, inv(12)(q12.q21). His mother's karyotype was 46, XX. His father had a karyotype of mos 46, XY/46, XY, inv(12)(q12q21) of 74∶6 cells in lymphocytes, without phenotype. A highly accurate analysis of chromosome 12 in lymphocytes detected an inversion of 12q13.1–q21.2 and a deletion at 12q21.2–q21.3 (Figure S1).

### Array CGH analysis

**Figure 2:**
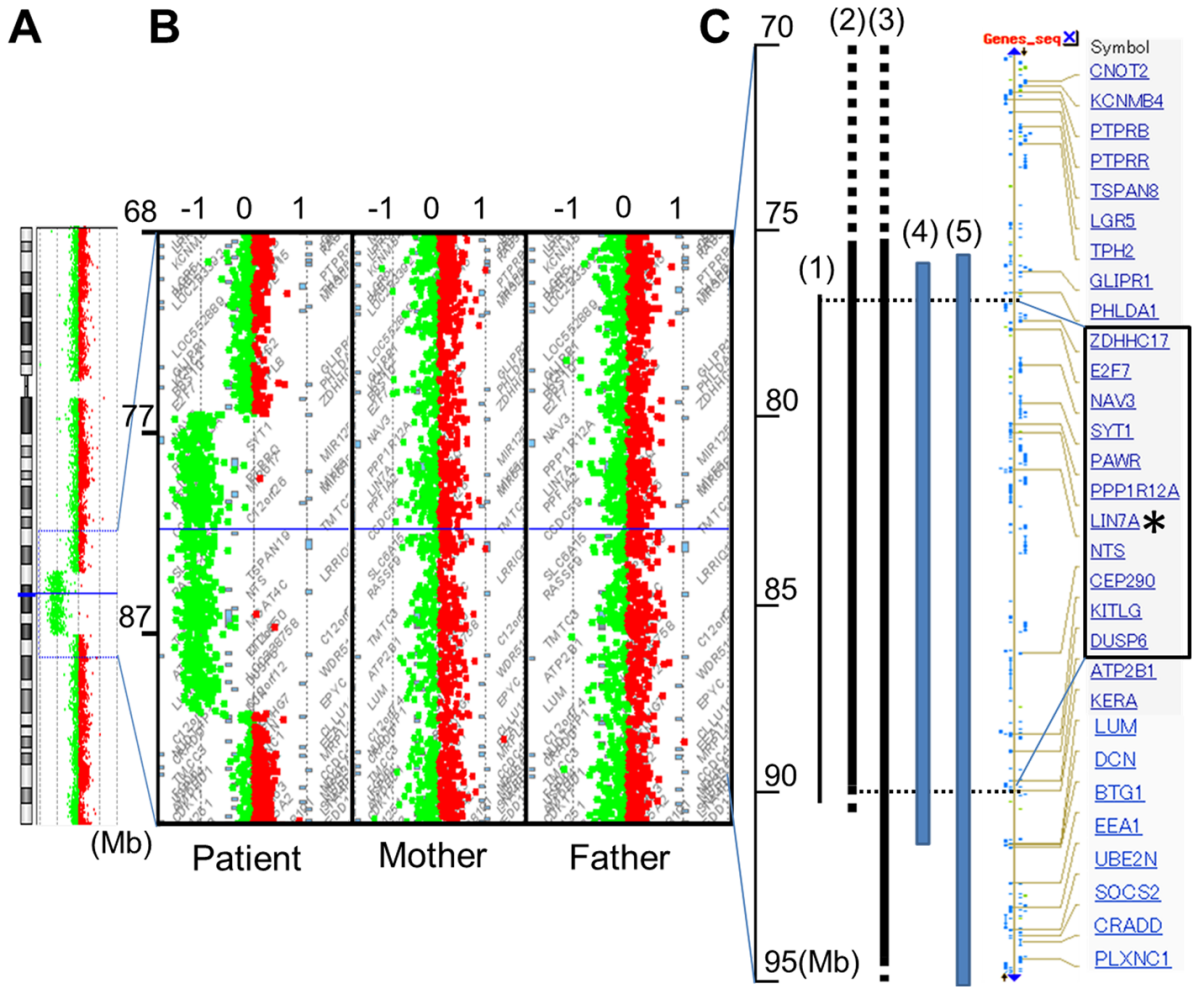
Array CGH analysis of chromosome 12. (**A**), Array CGH analysis of the entire chromosome 12 in the patient, showing a 14-Mb deletion of 12q21.2–q21.33 (77.2–91.2 M). (**B**), Detailed views of the microarray plots for the patient and his parents. The vertical axis shows megabases (Mb) from the telomere of 12q, and the horizontal axis shows the fold-change in copy number variation. (**C**), Comparison of the deleted positions. Bars indicate the deletion of the patient. (1) our patient, (2) Rauen, et al., 2002, (3) Klein, et al., (4) Brady, et al., and (5) Rauen, et al., 2000, respectively. The main genes existing in this area were listed as modified NCBI data. Genes surrounding *LIN7A*, from *ZDHHC17* to *DUSP6*, which are enclosed by the square, were included in the common deleted region from all five cases. An asterisk indicates *LIN7A*.

In the proband, CGH analysis using the Agilent Human genome CGH 180K array detected a 14 Mb deletion at 12q21.2–q21.33 (nt. 77 203 574–91 264 613) (Figure 2B). Therefore, the patient's karyotype was confirmed as 46, XY, del(12)(q21.2q21.33)inv(12)(q13.1q21.2). Array CGH analysis of his parents did not detect this 12q21 deletion (Figure 2B).

The gene encoding the zinc finger DHHC domain containing protein 17 *(ZDHHC17)* was disrupted at the break point of the deleted region at 77.2 Mb; a gene does not exist at the other break point at 91.3 Mb. Among the 72 genes located in this deleted region, several appeared as potentially important in relation to neuronal development and function, including *ZDHHC17*, neuron navigator 3 *(NAV3)*, synaptotagmin 1 *(SYT1)*, PRKC apoptosis WT1 regulator *(PAWR)*, *LIN7A*, etc. (Figure 2C). Here we focused on *LIN7A* as a candidate gene for ID and hypoplasia of the corpus callosum in this syndrome, and investigated its pathophysiological relevance.

### Developmentally regulated Lin-7 expression in the rat brain

**Figure 3:**
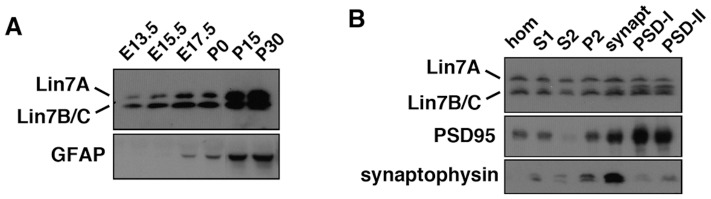
Expression profiles of Lin-7 proteins in the brain tissues. (**A**), Whole lysates (50 μg protein) of mouse cerebral cortices at various developmental stages were subjected to western blotting with antibodies against Lin-7 and GFAP. (**B**), Aliquots of brain fractions (10 μg) were immunoblotted with anti-Lin-7 (upper panel). hom, homogenate; S1, crude synaptosomal fraction; S2, cytosolic synaptosomal fraction; P2, crude synaptosomal pellet fraction; synapt, synaptosomal fraction; PSD-I, postsynaptic density fraction I; PSD-II, postsynaptic density fraction II. The blot was then re-probed with anti-PSD-95 (middle panel) and anti-synaptophysin (lower panel).

Although abnormal corticogenesis and synapse formation may contribute to the emergence of ID [22], Lin-7 expression has only been analyzed fragmentarily in the cerebral cortex during the developmental stage. Therefore, Lin-7 protein expression during embryonic and postnatal brain development was determined by western blot analysis. Lin-7A and Lin-7B/C were detected in the cerebral cortex from E13.5 to P30 with characteristic expression profiles (Figure 3A, upper panel). Lin-7A was first detected at E13.5, gradually increased during the embryonic stage, and then dramatically increased after P15, while the Lin-7B/C level increased gradually throughout the analyzed developmental stage. The developmental process was confirmed by visualizing the glial cell differentiation marker GFAP (Figure 3A, lower panel). These results suggest different roles of the Lin-7 isoforms during neuronal development.

Immunohistochemical staining and *in situ* hybridization further revealed that Lin-7A–C are differentially expressed in discrete populations of neurons throughout the adult mouse brain. Most neurons express only one Lin-7 isoform, although some cells contain two or even all three isoforms [23]. We then examined the subcellular fractionation of the cerebral cortex, and found that Lin-7A was comparably enriched in the presynaptic membranes, while Lin-7B/C tended to be present with a postsynaptic density (PSD) fraction (Figure 3B). Immunohistochemical analyses supported these findings, as Lin-7 was visualized in axons/dendrites of neurons in the thalamus, hippocampus CA1 region, and dentate gyrus in the adult rat brain [15]. These results indicate a possibility that Lin-7 isoforms have spatiotemporally specific roles during brain development and in synapse formation, maintenance, and functions.

### Role of Lin-7A in neuronal migration during corticogenesis

**Figure 4:**
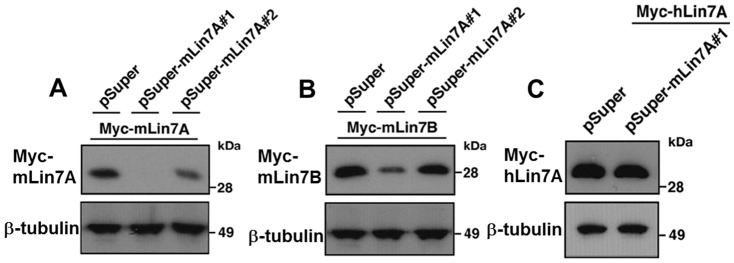
Characterization of RNAi vectors for Lin-7A. (**A**) pCAG-Myc-mLin-7A or (**B**) pCAG-Myc-mLin-7B was co-transfected into COS7 cells with control pSUPER vector, pSUPER–mLin7A#1, or pSUPER–mLin7A#2. After 48 h, cells were harvested and subjected to western blotting (20 μg protein per lane) with anti-Myc. Anti-β-tubulin was used as a loading control. (**C**), pCAG-Myc-hLin-7A was co-transfected into COS7 cells with control pSUPER vector or pSUPER–mLin7A#1. Analyses were done as in **A**.

To investigate whether *LIN7A* deletion may induce abnormal cerebral cortex cytoarchitecture leading to ID, we performed RNAi experiments, and examined the role of Lin-7A in the migration of newly generated cortical neurons during brain development. We designed two RNAi vectors – pSUPER–mLin7A#1 and pSUPER–mLin7A#2 – against distinct regions in the *mLin-7A* coding sequence. Both vectors efficiently knocked down mLin-7A expressed in COS7 cells (Figure 4A).

Since mLin-7B has been shown to be abundant in the cerebral cortex [23], we next investigated whether these RNAi vectors knocked down Lin-7B. Our results showed that mLin-7B was partially silenced by pSUPER–mLin7A#1, and hardly silenced by pSUPER–mLin7A#2 (Figure 4B). These results were expected based on the target sequence differences, and suggest that Lin-7B activity was partially maintained with the use of pSUPER-mLin7A#1, while almost completely maintained with pSUPER-mLin7A#2.

**Figure 5:**
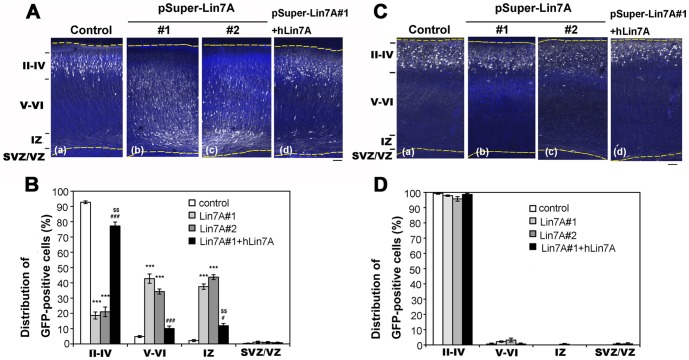
Role of Lin-7A in neuronal migration during corticogenesis. **(A)** and **(C)**, pCAG-EGFP was co-electroporated with (a) pSUPER vector (Control), (b) pSUPER–mLin7A#1, or (c) pSUPER–mLin7A#2 into cerebral cortices at E14.5, and fixed at P2 (**A**) or P7 (**C**). Coronal sections were prepared and immunostained with polyclonal anti-GFP (white). Nuclei were stained with DAPI (blue). (**A**, d) Rescue of pSUPER–mLin7A#1-induced migration defects. pSUPER-mLin7A#1 was electroporated with pCAG-GFP and pCAG-Myc-hLin-7A into cerebral cortices at E14.5, followed by fixation at P2. Analyses were performed as described above. Bar, 100 μm. (**B**) and **(D)**, Quantification of the distribution of GFP-positive cells in distinct regions of the cerebral cortex for each condition shown in **A** (**B**) and **C** (**D**). Values indicate the mean ± S.E.M. n = 3 each. ***P<0.001 vs. Control (Fisher's LSD test); ^#^ *P*<0.05, ^###^ *P*<0.001 vs. Lin7A#1 (Fisher's LSD test); ^$$^ *P*<0.01 vs. Control (Fisher's LSD test).

In the next set of experiments, the RNAi vectors and pCAG–EGFP were co-electroporated into progenitor cells in the ventricular zone (VZ) in the brains of embryonic mice, using *in utero* electroporation. Localization of transfected cells and their progeny was visualized at P2. In control experiments, vector-transfected neurons migrated normally to the superficial layer (layers II–IV) of the cortical plate (CP) (Figure 5A(a)). In contrast, a considerable portion of cells, which were transfected with pSUPER–mLin7A#1 or pSUPER–mLin7A#2, remained in the lower zone of the CP and the intermediate zone (IZ) (Figure 5A(b), (c)).

One-way ANOVA revealed significant effects of both vector injections [layers II–IV (F2,6 = 333.167, *P*<0.001); layers V–VI (F2,6 = 95.746, *P*<0.001); and IZ (F2,6 = 248.152 *P*<0.001)]. However, no significant effect was noted in the subventricular zone SVZ/VZ (F2,6 = 0.8, *P* = 0.4919). *Post-hoc* tests detected significant abnormal neuronal migration in cells that were injected with pSUPER–mLin7A#1 and #2 compared to control vector.

We next performed rescue experiments to rule out off-target effects. For this purpose, we used hLin-7A since it was resistant to pSUPER–mLin7A#1-mediated silencing (Figure 4C). When pCAG–EGFP and pSUPER–mLin7A#1 were co-electroporated together with pCAG–Flag-hLin7A, the positional defects by Lin-7A knockdown were rescued at P2 (Figure 5A(d)).

One-way ANOVA revealed significant effects of co-transfection with pCAG–Flag-hLin7A [layers II–IV (F2,6 = 364.775, *P*<0.001); layers V–VI (F2,6 = 103.885, *P*<0.001); and IZ (F2,6 = 176.481, *P*<0.001)]. However, no significant effects were observed in SVZ/VZ (F2,6 = 0.892, *P* = 0.4578). *Post-hoc* tests detected a significant rescue effect against abnormal neuronal migration. We then analyzed the effects of Lin-7A knockdown on the neuronal migration at P7. It should be noted that Lin7A-deficient cells reached the target location (layers II–IV) at this time point, indicating that silencing of Lin-7A delayed, but did not prevent, radial migration of cortical neurons (Figure 5C, D).

### Lin-7A is not involved in the cell cycle of neuronal progenitor cells

Since a prolonged cell cycle is known to result in delayed neuronal migration [24], we next asked whether Lin-7A silencing affected the proliferation of neurons produced in the VZ. We examined the impact of Lin-7A silencing on cell proliferation in the VZ by labeling S-phase cells with 5-ethynil-2′-deoxyuridine (EdU) to detect DNA replication. We found that Lin-7A-deficient cells were able to enter S-phase to a similar extent as the control pSUPER-transfected cells (Figure S2A, B). Thus, the cell cycle G1-progression rate did not statistically differ between the control and Lin-7A-deficient cells, implying that Lin-7A knockdown did not affect cell division/proliferation at VZ and SVZ. Furthermore, the positioning of the EdU/EGFP double-positive cells within VZ and SVZ was not affected by Lin-7A knockdown (Figure S2A). Overall, we conclude that the cell cycle was not affected in Lin-7A-deficient cells, and that neuronal positioning defects by Lin-7A knockdown are attributable to an abnormality in cell migration rather than cell proliferation.

### Role of Lin-7A in the interhemispheric connection of cortical neurons *in vivo*

Lin-7A deficiency could potentially affect not only cortical neuron migration but also axon path-finding, growth, and network formation during corticogenesis. Therefore, we analyzed interhemispheric axon projections of Lin-7A-deficient cortical neurons. To this end, we silenced Lin-7A in VZ progenitor cells at E14.5, and visualized axons in the contralateral hemisphere at P7.

**Figure 6:**
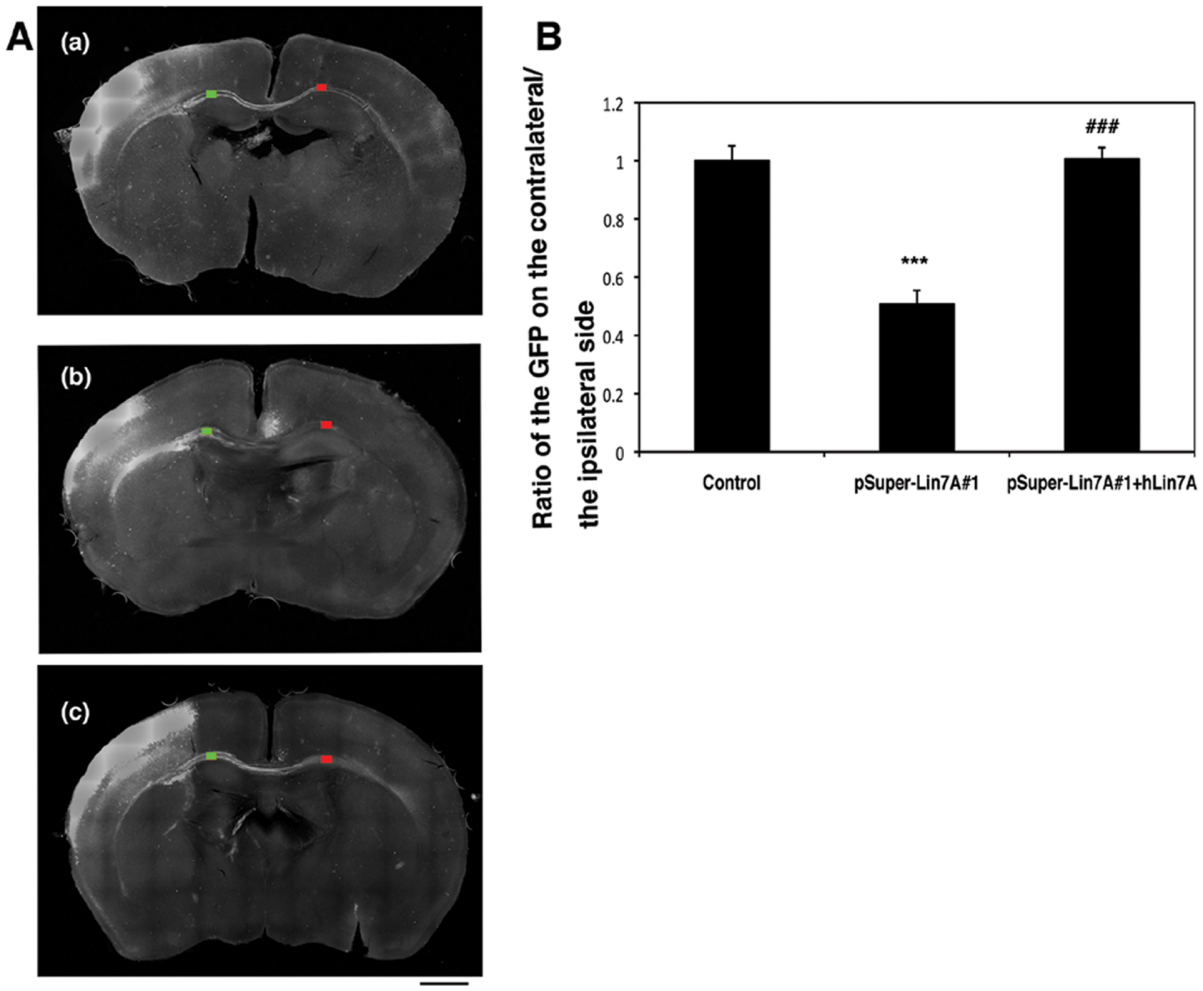
Role of Lin-7A in axon growth *ex vivo*. (**A**), Lin-7A deficiency affects the cortical axon growth. pCAG-EGFP was co-electroporated with (a) control pSUPER vector, (b) pSUPER-mLin7A#1, or (c) pSUPER-mLin7A#1+ hLin-7A into cerebral cortices at E14.5, and fixed at P7. Coronal sections were prepared and immunostained with polyclonal anti-GFP (white). Bar, 1 mm. (**B**), Quantitative analyses of the ratio of the intensity of GFP-positive axons in the area (green) of Lin-7A-deficient ipsilateral cortex to that in the area (red) of contralateral cortex in **A**. In the rescue experiments, hLin-7A was co-transfected as an RNAi-resistant version. Values indicate the mean ± S.E.M. n = 3 each. ***P<0.001 vs. Control (Fisher's LSD test); ^###^ *P*<0.001 vs. Lin7A#1 (Fisher's LSD test).

We found that, compared to the control cells, approximately half of the axons of Lin-7A-deficient neurons were disrupted from entering the contralateral hemisphere after leaving the corpus callosum (Figure 6A(a), (b), B). This phenotype was rescued by exogenous expression of hLin-7A (Figure 6A(c), B). These results suggest that Lin-7A is required for axon growth of excitatory neurons from the ipsilateral cortex to the contralateral cortex, the disturbance of which leads to hypoplasia of the corpus callosum.

## Discussion

The clinical features of 12q21 deletion that were shared by the four previously reported patients [1]–[4] and our patient include ID, low-set ears, sparse hair, prominent forehead, and hyper- or hypo-telorism (Table S1). Additionally, short upturned nose, small mandible, ocular abnormality, cardiac abnormality, and chronic dermatitis were each observed in three to four cases. Neuroimaging detected ventriculomegaly in three cases [1], [3], delayed myelination in one case [1], and mild hypoplasia of the corpus callosum in our present case. Our present case also involved sleep disturbance. These symptoms and signs, which we observed in this patient, can be considered phenotypes of 12q21 deletion, since there have only been a few reported patients, and the features of 12q21 deletion have not been established.

The previously reported cases of 12q21 deletion were analyzed by G-banding [1], [4] and array CGH with BAC clones [2], [3]. The deletion in the present case, from 12q21.2 to 12q21.33 (77.2–91.2 Mb), was the shortest and most definitively detected deletion in any of the five cases, suggesting that this region contains the gene(s) responsible for the concordant features (Figure 2C). Our patient also had the inversion of 12q13.1–q21.2. ZDHHC17 was disrupted on one side of the deletion, while no gene exists on the other side of the deletion. Information about the other end of inversion was not obtained; we do not deny the possibility that the disrupted ZDHHC17 or putative gene on the other side of the inversion could have gained some function in addition to the loss of function.

The present study provided evidence that functional defects of Lin-7A may be involved in the pathophysiology of 12q21-deletion syndrome, possibly through impaired cerebral development. Although molecular mechanisms governing the pathophysiology are enigmatic, we assume that Lin-7A participates in neuronal migration during corticogenesis. It should be noted here that the migration delay of Lin-7A-deficient neurons at P2 was recovered at P7, and these cells eventually arrived at their correct destination.

While migration abnormality has not been reported in the 12q21 deletion syndrome. It is, however, likely that the delayed migration process *per se* may cause deficiency in neuronal functions, leading to ID in 12q21-deletion syndrome. Interestingly, comparable phenotypes have been observed in the course of pathophysiological analyses of *SIL1*, a causative co-chaperone gene of Marinesco-Sjogren syndrome, in which ID is a major symptom [25].

Characteristic phenotypes were not observed in the cerebral cortex of Lin-7A/B-knockout mice [23]. Notably, up-regulation of Lin-7C was observed in the knockout mouse, and, thus, may compensate for the knockout phenotype. On the other hand, acute knockdown by *in utero* electroporation is suggested to circumvent the compensatory effects of general gene-knockout approaches. In this context, a scaffold protein Doublecortin- or a cytoskeleton-related molecule, Sept4-deficient mice also exhibited no obvious morphological alteration in the cerebral cortex [26], [27], while acute knockdown of these genes by *in utero* electroporation resulted in defective neuronal migration [21], [28].

The corpus callosum was hypoplastic in our present 12q21-deletion syndrome case. The corpus callosum consists of more than 200 million axons, which originate from neurons of layers II, III, and V of the cerebral cortex [29], [30]. Given that axons from the hemisphere containing Lin-7A-deficient neurons did not efficiently extend into the contralateral cortex, the hypoplasia of the corpus callosum in our proband is likely attributable to defects in Lin-7A function.

*LIN7A* is known to relate to synaptic functions [10], [11]. Here we clarified that Lin-7A was enriched in synapses, and played a pivotal role in cortical neuron migration and axon network formation during corticogenesis. Although neuronal migration defects were temporal, and neurons eventually located at their correct positions, loss of function of Lin-7A should cause a subtle abnormal architecture of the developing cerebral cortex, leading to dysfunction of synapses and/or axon growth. Overall, our results indicate that the ID and hypoplasia of the corpus callosum observed in our 12q21-deletion syndrome case may be the phenotype of *Lin7A* deletion.

## Supporting Information

Figure S1 **Chromosome 12 analysis by a highly accurate technique.** Arrows indicate the inversion of q13.1q21.2. The blue line next to the inversion area indicates the deletion of q21.2q21.3.
(TIF)

Figure S2 **Effects of Lin7A silencing on the DNA replication in S-phase of the cell cycle.** (**A**), E14 cortices were co-electroporated with pCAG-EGFP together with control pSUPER vector or pSUPER–mLin7A#1. Coronal sections were visualized for GFP (green) and EdU (red). Arrowheads indicate EdU/GFP double-positive cells. Dotted lines represent ventricular surface. Bars, 50 μm. (**B**), Quantification of EdU/EGFP double-positive cells among EGFP-positive cells. Values indicate the mean ± S.E.M. n = 3 each.
(TIF)

Table S1 **Clinical features of 12q21 deletion syndrome.**
(DOCX)
