# Supplementary figures and images for: LIN7A Depletion Disrupts Cerebral Cortex Development, Contributing to Intellectual Disability in 12q21-Deletion Syndrome
Source: PLoS One. 2014 Mar 21;9(3):e92695. doi: 10.1371/journal.pone.0092695 (PMC3962435; doi:10.1371/journal.pone.0092695)

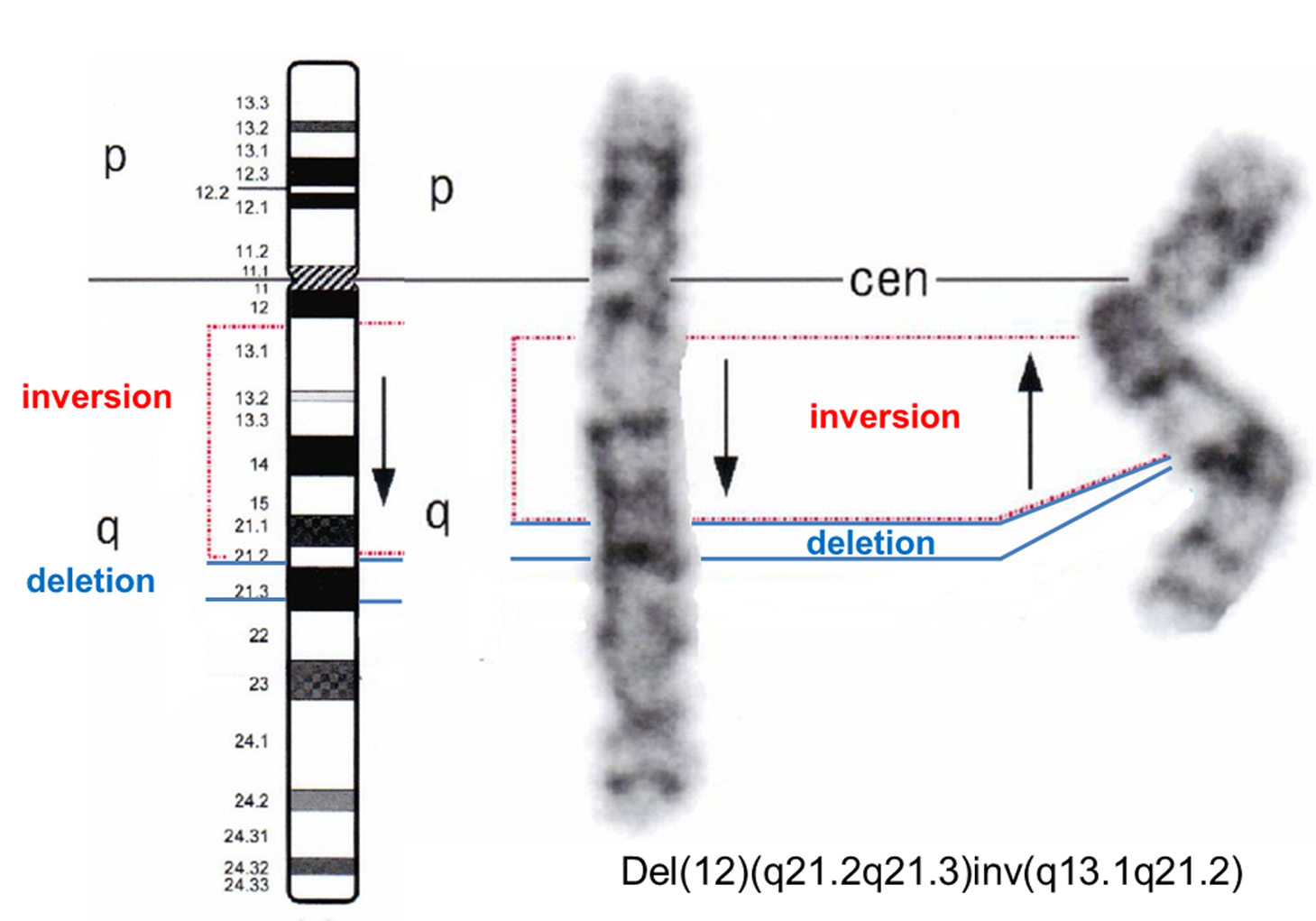

Supplement: Figure S1 — Chromosome 12 analysis by a highly accurate technique. Arrows indicate the inversion of q13.1q21.2. The blue line next to the inversion area indicates the deletion of q21.2q21.3. (TIF) [file pone.0092695.s001.tif]

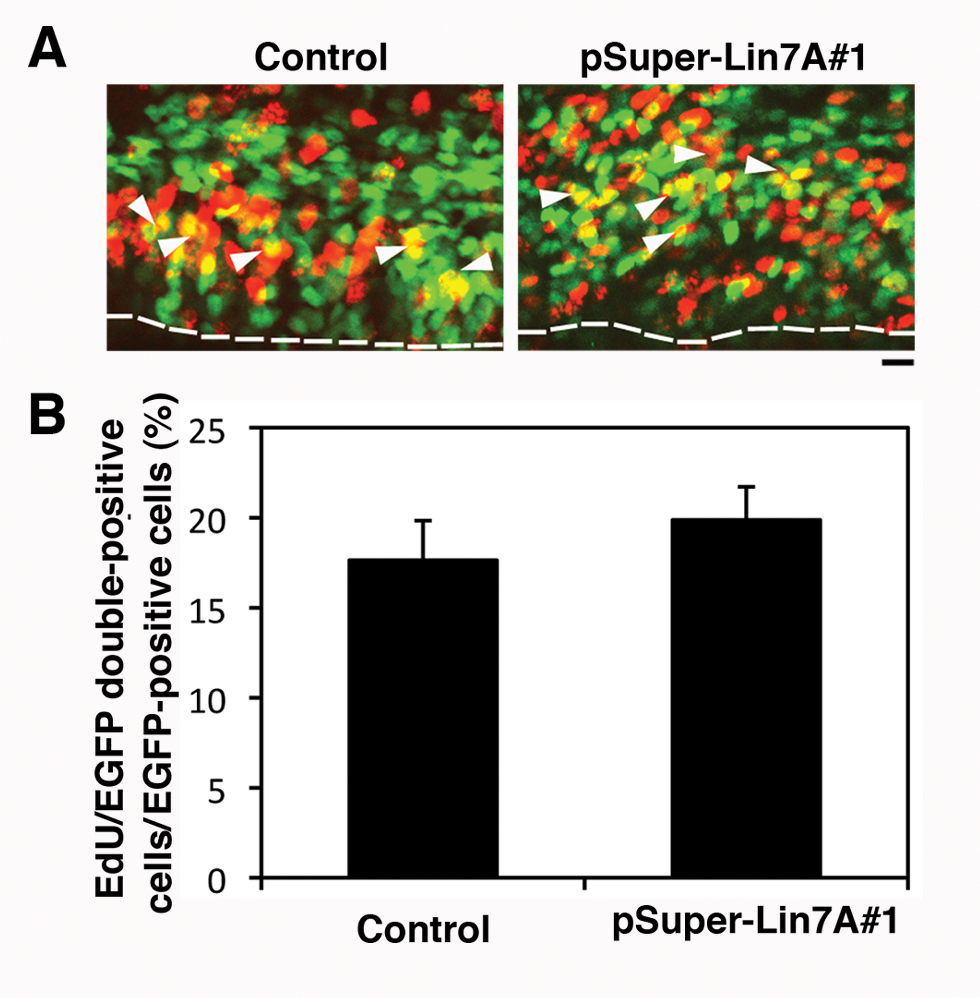

Supplement: Figure S2 — Effects of Lin7A silencing on the DNA replication in S-phase of the cell cycle. (A), E14 cortices were co-electroporated with pCAG-EGFP together with control pSUPER vector or pSUPER–mLin7A#1. Coronal sections were visualized for GFP (green) and EdU (red). Arrowheads indicate EdU/GFP double-positive cells. Dotted lines represent ventricular surface. Bars, 50 μm. (B), Quantification of EdU/EGFP double-positive cells among EGFP-positive cells. Values indicate the mean ± S.E.M. n = 3 each. (TIF) [file pone.0092695.s002.tif]
