# Supplementary material for: LIN7A Depletion Disrupts Cerebral Cortex Development, Contributing to Intellectual Disability in 12q21-Deletion Syndrome
Source: PLoS One. 2014 Mar 21;9(3):e92695. doi: 10.1371/journal.pone.0092695 (PMC3962435; doi:10.1371/journal.pone.0092695)
Supplement: Table S1 — Clinical features of 12q21 deletion syndrome. (DOCX) [file pone.0092695.s003.docx]

**Table S1.** Clinical features of 12q21 deletion syndrome

|  | Brady et al.  1999 | Rauen et al.  2000 | Rauen et al.  2002 | Klein et al.  2005 | Present case |
| --- | --- | --- | --- | --- | --- |
| Cytogenetic  Karyotype | 46,XX,del(12)  (q21.2q23.2) | 46,XX,del(12)  (q21.2q22) | 47,XYY,del(12)  (q21.1q21.33) | 46,XY,del(12)  (q21.1q22) | 46,XY,del(12)(q21.2q21.33),  inv(12)(q13q21) |
| Sex | Female | Female | Male | Male | Male |
| Intellectual disability | + | + | + | + | + |
| Sparse hair | + | + | + | + | + |
| Prominent forehead | + | + | + | + | + |
| Hypo-/hyper-telorism | Hyper | Hyper | Hypo | Hyper | Hypo |
| Ocular abnormality | − | + | − | + | + |
| Downslanting palpabral fissures | − | + | + | − | − |
| Short upturned nose | − | + | + | + | + |
| Small mandible | No report | + | + | No report | + |
| Low-set ears | + | + | + | + | + |
| Skin findings | No report | Hyperkeratotic eruption | Hyperkeratotic eruption | Dermatitis | − |
| Cardiac abnormality | − | small VSD | PDA | PDA | − |
| Cranial MRI | No report | Ventriculomegaly  Delayed myelination | − | Ventriculomegaly | Ventriculomegaly  Hypoplasia of the corpus callosum |

VSD: ventricular septal defect; PDA: Patent ductus arteriosus.
